# Single-Crystalline Si Stacked AlGaN/GaN High-Electron-Mobility Transistors with Enhanced Two-Dimensional Electron Gas Density

**DOI:** 10.3390/mi16111214

**Published:** 2025-10-25

**Authors:** Goeun Ham, Eungyeol Shin, Sangwon Yoon, Jihoon Yang, Youngjin Choi, Gunwoo Lim, Kwangeun Kim

**Affiliations:** 1 Department of Electronics and Information Engineering, Korea Aerospace University, Goyang 10540, Republic of Korea; hamgoeun456@kau.kr; 2 Interdisciplinary Program in Space Systems Engineering, Korea Aerospace University, Goyang 10540, Republic of Korea; shineg0211@kau.kr; 3 Department of Semiconductor Science, Engineering and Technology, Korea Aerospace University, Goyang 10540, Republic of Korea; lgwkkk@kau.kr; 4 School of Electronics and Information Engineering, Korea Aerospace University, Goyang 10540, Republic of Korea; steve0829@kau.kr (S.Y.); johnny1835@naver.com (J.Y.); tp4ryn7k@gmail.com (Y.C.)

**Keywords:** AlGaN/GaN HEMT, stacking, single-crystalline Si, 2DEG

## Abstract

High-electron-mobility transistors (HEMTs) are characterized by the formation of a two-dimensional electron gas (2DEG) induced by the polarization effects. Considerable studies have been conducted to improve the electrical properties of HEMTs by regulating the 2DEG density. In this study, a Si/GaN heterojunction was fabricated through the transfer of a heavily boron-doped Si nanomembrane. The holes in the p-Si layer integrated on top of the HEMT not only increased the surface positive charge, which eventually increased the density of electrons at the AlGaN/GaN interface, but also acted as a passivation layer to improve the performance of AlGaN/GaN HEMTs. Electrical characterization revealed that the maximum drain current increased from 668 mA/mm to 740 mA/mm, and the maximum transconductance improved from 200.2 mS/mm to 220.4 mS/mm. These results were due to the surface positive charge induced by the p-Si layer, which lowered the energy band diagram and increased the electron concentration at the AlGaN/GaN interface by a factor of 1.4 from 1.52 × 10^20^ cm^−3^ to 2.11 × 10^20^ cm^−3^.

## 1. Introduction

Recently, GaN has attracted attention as a material for devices used in high-voltage and high-frequency environments owing to its wide bandgap [1,2,3,4]. In particular, in the case of AlGaN/GaN high-electron-mobility transistors (HEMTs), a two-dimensional electron gas (2DEG) channel formed by the polarization effect of AlGaN and GaN has the advantage of being able to operate at radio frequency. Research on achieving stable devices under high-voltage and high-frequency conditions is actively pursued by reducing surface defects and controlling the 2DEG density. In this context, analysis of 2DEG density variations induced by surface treatments of the HEMT upper layer is currently being conducted [5,6,7,8,9].

It has often been studied that the variation of 2DEG density can be controlled by adjusting the thickness and doping concentration of the GaN cap layer [10,11,12]. It was observed that, when the GaN cap layer was excessively thick, the conduction band rose, hindering the formation of the 2DEG channel and limiting the doping effect [13,14,15]. The parasitic 2DEG channel formed at the SiN/GaN cap layer interface could increase the current, but at the same time, the 2DEG density at the AlGaN/GaN interface decreased and device degradation occurred [5,15]. In the case of using Mg-doped p-GaN as a cap layer, not only is it difficult to achieve high doping concentration, but the high activation energy of Mg limits the hole concentration that can be supplied [16]. Furthermore, the Mg diffused into the AlGaN barrier layer during processing can be ionized and formed acceptor-like traps [17,18,19]. Due to the lower activation energy of boron, the boron-doped Si can exhibit higher doping efficiency than Mg when used as a hole injector. Currently, research on using doped Si deposited on top of HEMTs to improve the current properties has not been sufficiently conducted. The biggest reason for this is that the lattice mismatch between Si(100) and GaN(002) generates strain, which can degrade the device performance [20].

In the formation of heterostructures, the heteroepitaxy method is impeded by significant lattice mismatch, which can lead to the formation of dislocations and the degradation of crystalline order. Furthermore, wafer bonding is susceptible to defect formation or even failure during subsequent thermal processing due to mismatched thermal expansion coefficients. As an alternative to these conventional methods, NM stacking has been proposed as a technology to effectively integrate materials with disparate physical properties. The key advantage of the NM stacking technique lies in the flexible mechanical properties of the ultrathin membrane, which effectively accommodates the strain energy arising from material mismatches without causing structural failure. Additionally, a thermodynamically stable interlayer formed at the bonded interface mitigates the atomic-scale lattice mismatch, enabling the formation of a stable heterojunction [21,22].

In this study, a HEMT was fabricated by transferring heavily doped Si using a nanomembrane (NM) transfer method. Compared with the epitaxially grown Si/GaN heterostructure, the NM printing method enables the formation of Si/GaN stacked heterojunction without strain induced by the lattice mismatch. Moreover, when using boron-doped Si, the low activation energy of dopant enables efficient hole supply into the GaN. Through energy band diagram simulations, we observed that stacking the p-Si layer lowered the energy band at the AlGaN/GaN interface, increasing the 2DEG density. I-V characteristics revealed improved electrical properties of the p-Si stacked HEMT.

## 2. Methods

The heavily boron-doped silicon-on-insulator (SOI, N_A_ = 5 × 10^19^ cm^−3^) wafer was employed to produce a crystalline Si NM. The thickness of the top p-Si layer of the SOI was 200 nm and that of sacrificial SiO_2_ layer was 1 μm. Etching holes were patterned on the surface of SOI for the undercut process, which allowed etching the SiO_2_ through the holes when immersed in an HF solution.

**Figure 1:**
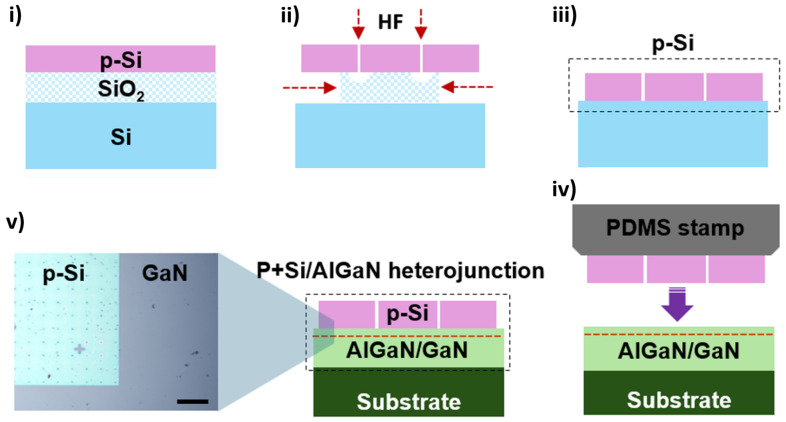
Fabrication process of the AlGaN/GaN High-electron-mobility transistors (HEMT) with p-Si stacking. (**i**) A heavily boron-doped silicon-on-insulator (SOI) wafer is patterned, and (**ii**) immersed in HF solution to obtain a p-Si nanomembrane (NM). (**iii**,**iv**) The p-Si NM is then transferred onto the HEMT wafer using a polydimethylsiloxane stamp. (**v**) The optical image of stacked Si/GaN heterojunction is shown (scale bar = 100 μm). The SOI wafer has a 50 μm spaced hole pattern that was etched to accelerate the HF removal of the SiO_2_ layer. To form the junction between the Si NM and GaN, thermal annealing was performed at 600 °C for 3 min.

**Figure 2:**
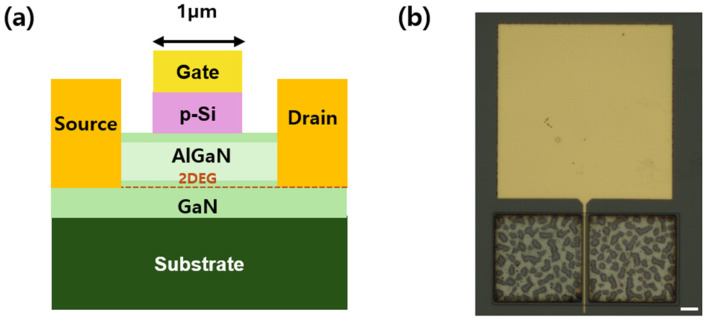
(**a**) Cross-sectional schematic of the Si stacked HEMT with a gate length of 1 μm and (**b**) optical top view of the device with W_gate_/L_gate_ ratio of 5 (scale bar = 10 μm). The electrode on the Si was deposited as a Schottky metal.

Figure 1 illustrates the fabrication process of the Si NM stacked HEMT utilizing the NM printing technique. The top p-Si layer obtained by undercutting the SiO_2_ sacrificial layer of the SOI using a diluted HF solution was separated from the wafer (i and ii). The HEMT devices were epitaxially grown on a sapphire substrate via metal–organic chemical vapor deposition, featuring an Al molar fraction of 0.3 in the AlGaN layer, with a GaN cap layer of approximately 2 nm on top of the AlGaN. The structures consist of a 5 µm-thick GaN buffer layer, a 20 nm-thick AlGaN barrier layer. The p-Si NM obtained through undercutting (iii) was subsequently transferred onto the grown HEMT wafer (iv). Annealing was performed at 600 °C for 3 min in Ar ambient to achieve the Si/GaN stacked heterostructure (v). During this process, the thin Si layer expanded to match the lattice constant of GaN, leading to the formation of an intermediate layer at the Si/GaN interface. According to previous study [20], when two materials with a large lattice constant difference, such as Si and GaN, are joined, a thin amorphous layer with lower energy and greater thermodynamic stability than the original crystal structures is formed to relieve the mismatch between the two lattices. The TEM analysis revealed that the thickness of the interlayer was less than 2 nm. The resulting Si/GaN stacked junction was found to alleviate the lattice mismatch issue. As illustrated in Figure 2, the Schottky gate region (1 × 50 μm^2^) was deposited with Al on the Si layer. Finally, the electrical characteristics of the fabricated devices were analyzed in detail using a Keithley 4200-SCS semiconductor parameter analyzer (Solon, OH, USA).

## 3. Results and Discussion

A single-crystalline Si NM with a hole pattern was released from a SOI wafer and was transferred onto via transfer printing the top surface of the HEMT using a polydimethylsiloxane (PDMS) stamp. The transfer process was controlled by a kinetically controlled adhesion mechanism based on the viscoelastic properties of the PDMS stamp. The adhesion strength between the stamp and the membrane increases monotonically with the peel velocity V. A transition between pick-up and printing occurs under the condition where the energy release rates of the two interfaces become balanced. When the stamp is peeled from the substrate at a constant velocity, the interfacial energy release rate G is related to the peel force F and sample width W as follows,

(1) $$G=\frac{F}{w},$$

which includes both the interfacial bond-breaking energy and the viscoelastic dissipation near the crack tip. When G reaches the critical energy release rate according to the Griffith criterion of fracture mechanics, the crack propagates steadily.

The critical energy release rate of the stamp/membrane interface exhibits a velocity dependence and can be expressed as

(2) $$G_{crit}^{stamp/film}=G_{crit}^{stamp/film}\left(V\right),$$

The critical peel forces for the pick-up state, where the membrane is detached from the substrate and the printing state, where the membrane is separated from the stamp are given by

(3) $$F_{pickup}=wG_{film/substrate}^{crit},$$

(4) $$F_{printing}=wG_{stamp/film}^{crit}\left(V\right),$$

The critical velocity $V_{c}$, at which the two interfacial energy release rates become equal is defined as

(5) $$G_{film/substrate}^{crit}=G_{stamp/film}^{crit}\left(V_{c}\right),$$

According to this relation, for V > $V_{c}$, the viscoelastic dissipation enhances the interfacial adhension at the stamp/membrane interface, resulting in the membrane being picked up by the stamp. In contrast, V < $V_{c}$, the interfacial adhension at the stamp/membrane interface becomes relatively weak, causing the membrane to remain on the substrate.

Since the transfer process using PDMS stamps is kinetically controlled, the temperature plays an important role. The temperature dependence can be expressed using the temperature shift factor $a_{T}$.

(6) $$G_{crit}^{stamp/film}=G_{crit}^{stamp/film}\left(Va_{T}\right),$$

As the temperature increases, $a_{T}$ decreases, leading to a higher $V_{c}$.

Based on this theoretical framework, transfer printing was performed in this study by bringing the PDMS stamp into contact with the top surface of the HEMT and subsequently peeling it off under conditions that satisfy the kinetically controlled transfer regime.

**Figure 3:**
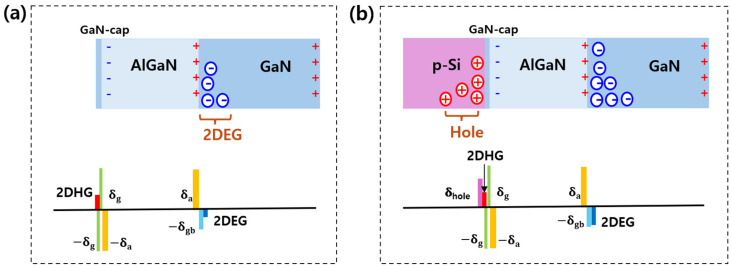
Charge neutrality at thermal equilibrium for (**a**) conventional HEMT and (**b**) p-Si stacked HEMT. When the p-Si layer is bonded, hole charges accumulate on the GaN cap surface, and the 2DEG density increases due to the charge neutrality condition. Since the GaN buffer layer is sufficiently thick, the charges on the substrate side can be neglected.

Figure 3 shows the charges accumulated at the interfaces in conventional and Si stacked HEMTs. In the case of the GaN buffer polarization charge (δ_gb_), the thickness of the GaN layer is greater than 5 µm and the positive charges on the substrate side can be neglected. The polarization charge of AlGaN is denoted as δ_a_ and the polarization charge of GaN is denoted as δ_g_. In the conventional HEMT, the two-dimensional hole gas (2DHG) and 2DEG are generated by the polarization charges of AlGaN and GaN. When the p-Si layer is stacked, holes (δ_hole_) accumulate on the GaN cap surface. At thermal equilibrium the charge in the AlGaN region is required to satisfy the charge neutrality condition, which can be expressed as $\delta_{hole}+2DHG-\delta_{gb}-2DEG=0.$ Therefore, the 2DEG increases when holes move from the p-Si layer to the GaN cap and accumulate as positive charges [23,24,25]. Furthermore, this can be explained by the fact that the electric field induced by the positive charges accumulated on the GaN surface mitigates the electric field within the AlGaN barrier, thereby influencing the 2DEG density.

**Figure 4:**
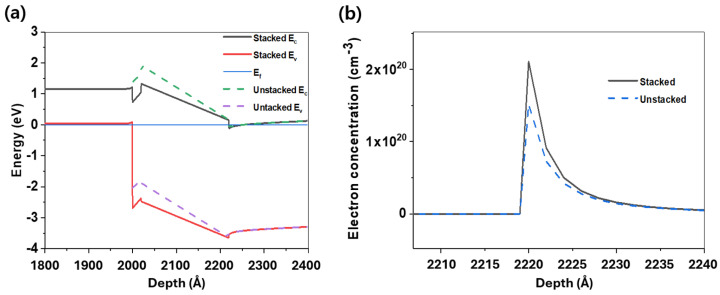
(**a**) Energy band diagram change when a p-Si layer is stacked, obtained using a band engineering simulator, and (**b**) maximum electron concentration in the 2DEG. The thickness of Si was set to 200 nm, and N_a_ = 5 × 10^19^ cm^−3^. For comparison with conventional HEMTs, the front and back bands are omitted. Although the intermediate layer formed by the Si/GaN junction was not considered, the presence of the p-Si layer causes the AlGaN/GaN energy band to shift downward, resulting in an increase in the 2DEG density. The increase in 2DEG density is supported by simulation results showing a 1.4-fold increase in the electron concentration at the AlGaN/GaN interface.

Figure 4 illustrates the energy band diagrams and the changes in electron concentrations with and without p-Si layer, developed by a BandEng simulator. The holes accumulated at the p-Si/GaN cap interface cause the energy band of the upper AlGaN layer to bend downward. Furthermore, when a p-Si layer with a doping concentration of 5 × 10^19^ cm^−3^ is bonded to the AlGaN/GaN HEMT, the band alignment causes the energy band at the AlGaN/GaN interface to bend downward, resulting in an increased 2DEG density. Compared to that of a conventional HEMT, the electron concentration of the HEMT with the stacked p-Si layer increased by a factor of 1.4 from 1.52 × 10^20^ cm^−3^ to 2.11 × 10^20^ cm^−3^.

**Figure 5:**
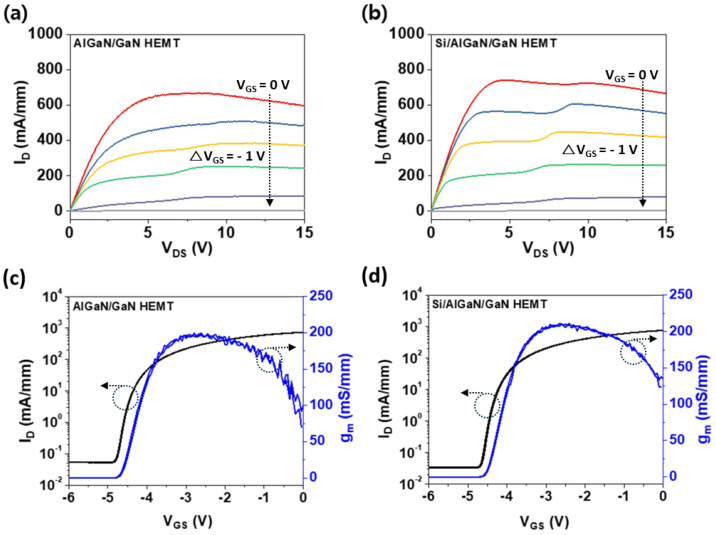
I_D_–V_DS_ characteristics of (**a**) a conventional HEMT and (**b**) a Si stacked HEMT, measured with V_GS_ ranging from 0 to −5 V in −1 V steps. The incorporation of the p-Si layer increased the maximum drain current from 668 mA/mm to 740 mA/mm. I_D_–V_GS_ characteristics of (**c**) a conventional HEMT and (**d**) a Si stacked HEMT. The maximum transconductance (g_m_) increased from 200.2 mS/mm to 220.4 mS/mm, and the threshold voltage (V_t_) shifted from −4.24 V to −4.01 V.

The maximum drain current showed an increase from 668 mA/mm to 740 mA/mm when the p-Si was stacked (Figure 5a,b). The increase in the maximum drain current is related to the enhanced 2DEG density as most of the boron dopants in the p-Si are ionized and act as negative charges at the Si/GaN interface, thereby lowering the energy band and increasing the 2DEG density. The current-carrying capability of the HEMT can be mathematically represented as the product of the 2DEG mobility and the 2DEG sheet carrier density, which directly correlates with the maximum drain current [26,27,28]. These findings suggest that the increase in 2DEG density resulting from p-Si deposition is the primary driver behind the observed increase in maximum drain current. The HEMT stacked with p-Si demonstrates not only a reduction in leakage current within the subthreshold region, reduced to about 65% of its original value (from 0.054 to 0.035 mA/mm), but also an increase in maximum transconductance (g_m_) from 200.2 mS/mm to 220.4 mS/mm (Figure 5c,d). This enhancement can be attributed to the formation of Si/GaN stacked heterointerface that improved the transfer of positive charges and induced the reduction in traps [9,29].

The threshold voltage (V_t_) before and after the incorporation of p-Si was measured to be −4.24 V and −4.04 V, respectively, corresponding to a shift of approximately 0.2 V. In HEMTs, the positive shift of V_t_ is generally ascribed to variations in the 2DEG density [16,30]. Some studies have reported that while V_t_ shifts positively with increasing doping concentration of the cap layer, it tends to decrease again once the concentration exceeds a certain level [4,18,31]. When the heterointerface of Si and GaN are produced via heteroepitaxy, defects and strain arising from the lattice mismatch at the interface can deteriorate the electrical characteristics of the device. Excessive thickness of the Si can also lead to internal strain and dislocation. The Si NM used in this work had a relatively thin thickness of approximately 200 nm and the thin Si layer expands to match the lattice constant of GaN with no internal strain issue by employing the NM stacking method.

The p-Si was stacked to shift the V_T_ in the positive direction while simultaneously increasing the 2DEG density. Previous studies have deposited p-GaN to achieve a positive V_T_ shift; however, the 2DEG density generally decreased and the electrical characteristics were relatively degraded [18,32,33]. In this work, both conventional HEMTs and HEMTs stacked with p-Si were fabricated with Schottky metal gates to analyze their electrical characteristics. When Si was stacked, the effective work function of the gate metal increased, resulting in a positive shift in V_T_. This suggests a method for realizing normally off operation by stacking Si—previously uncommon as a gate material in HEMTs—on the top while maintaining the 2DEG density.

## 4. Conclusions

In this study, heavily doped p-Si was stacked on top of the AlGaN/GaN HEMT through the NM transfer method to improve the performance of HEMT. The holes accumulated at the p-Si/GaN interface require negative charges on the AlGaN/GaN surface to satisfy the charge neutral state in the thermal equilibrium state. The accumulated holes thus cause the downward energy band at the AlGaN/GaN interface, increasing the 2DEG density. Through the stacking of crystalline Si with the AlGaN/GaN HEMT, the maximum drain current increased from 668 mA/mm to 740 mA/mm and leakage current has been reduced by 65% of the conventional HEMT. Furthermore, Si, which is not typically employed as a gate material, can be integrated onto the upper region of the HEMT to shift the threshold voltage positively, while simultaneously enhancing the 2DEG density and thereby improving the device’s electrical characteristics. This improvement is attributed to the formation of Si/GaN stacked heterojunction as charge injector and passivation layer. This demonstrates the reliability of a device that integrates two semiconductors previously considered incompatible due to lattice mismatch, thereby contributing to the advancement of heterojunction-based devices.

## Data Availability

The original contributions presented in the study are included in the article, further inquiries can be directed to the corresponding author.
